# Solid Sirolimus Self-microemulsifying Drug Delivery System: Development and Evaluation of Tablets with Sustained Release Property

**DOI:** 10.22037/ijpr.2019.1100847

**Published:** 2019

**Authors:** Chun Tao, Xu Wen, Qian Zhang, Hongtao Song

**Affiliations:** a *Department of Pharmacy, Fuzong Clinical Medical College of Fujian Medical University (Fuzhou General Hospital), Fuzhou 350025, PR China.*; b *Department of Inorganic Chemistry, College of Pharmacy, Fujian Medical University, Fuzhou 350108, PR China.*

**Keywords:** Sirolimus, Self-microemulsifying drug delivery system, Tablet, Sustained release, Bio-equivalent

## Abstract

The clinical application of sirolimus (SRL) as an immunosuppressive agent is largely hampered by its narrow therapeutic range. This study focused on developing SRL tablets with a sustained release profile for better safety. SRL was highly water insoluble and its solubility has been efficiently enhanced by preparing self-microemulsifying drug delivery system (SMEDDS). The SRL-SMEDDS was physically adsorbed by microcrystalline cellulose (MCC). The sustained release of SRL was achieved by addition of hydroxypropyl methylcellulose (HPMC) to prepare tablets. The formulation of the tablets was optimized by single factor test and orthogonal design. The optimal formulation was composed of 10% of HPMC 100lv and 5% of HPMC K4M. The *in-vitro* release profiles of the optimal tablets were further investigated for the influence of hardness, shape, preparing method, release method, stirring speed, and medium. The release kinetic of SRL from the tablets was demonstrated to be erosion of HPMC. Pharmacokinetic study on beagle dogs showed that the SRL-SMEDDS tablets were bio-equivalent to the commercial tablets but lower C_max_ and larger T_max_ were achieved. In conclusion, the SMEDDS tablets were presented as promising delivery system for sustained release of SRL.

## Introduction

Sirolimus (SRL) has been widely used as an immunosuppressive agent for organ transplantation (1). But the narrow therapeutic range is one of the main barriers for the clinical application of SRL. The blood concentration of SRL is suggested to be 4-20 ng/mL for rejection of organ transplantation. When it is used together with ciclosporin, the recommended concentration is reduced to be 4-12 ng/mL (2). Most of the related side effects like hypertension, hyperlipemia, and cytopenias are dose dependent (3, 4). The commercial tablet of SRL (Rapamune^®^) exhibits a rapid drug release in 2 h, which is not able to maintain a steady blood concentration. To keep the blood concentration of SRL in the therapeutic range, a sustained release behavior is desired. 

The SRL belongs to the biopharmaceutics classification system (BCS) class II drug category, showing poor solubility and good permeability. Nanocrystal technology is applied to improve the solubility of SRL in the Rapamune^®^. But the SRL nanocrystals produced by ball milling are time-consuming, inefficient, and expensive. 

The self-microemulsifying drug delivery system (SMEDDS) presents as an excellent carrier for hydrophobic drugs (5). Compared with the nanocrystals prepared by ball milling, SMEDDS is a more economical and efficient method (6). The SMEDDS is also commercially available as it has been applied in the commercial products including ciclosporin (Sandimmun Neoral^®^), ritonavir (Norvir^®^), and saquinavir (Fortovase^®^). 

The SMEDDS is usually composed of oil, emulsifier, and co-emulsifier (7). After oral administration, the SMEDDS can form emulsions of nano-sized droplets upon body fluids in the gastrointestinal tract. It was reported that SMEDDS containing SRL achieved enhanced intestinal absorption, leading to higher bioavailability than the commercial oral solution and raw SRL powder (8). 

In our previous study, solid SRL-SMEDDS was developed and optimized to improve the stability of SRL during storage (9, 10). This study focused on preparation and characterization of the sustained release tablets using SRL-SMEDDS. 

## Experimental

*Materials*

SRL was purchased from Kerui (Fuzhou, China). Transcutol HP and Labrafil M 1944CS, Cremophor EL were gifted by Gattefossé (Brittany, France). Hydroxypropyl methylcellulose (HPMC100lv, K4M, K15M and K100M) was purchased from Colorcon (Shanghai, China). MCC (Avicel PH-101) was obtained from FMC (Philadelphia, US). Lactose (Foremost 315WG) was obtained from Kerry Group (Tralee, Ireland). All other reagents were of analytic grade. 

*Preparation of SMEDDS*

The SMEDDS was prepared based on our previous study (9). Briefly, 0.1 g of SRL was dissolved in Transcutol HP (1.92 g) before being mixed with Labrafil M 1944CS (2.24 g) and Cremophor EL (3.84 g) by stirring. Citric acid (16.2 mg) was added to prevent the degradation of SRL in the SMEDDS. 

*Characterization of the SMEDDS*

The emulsion was prepared by addition of 0.5 mL of SMEDDS into 50 mL of water under magnetic stirring at 37 °C for 10 min. The average diameter of emulsion droplets was measured by the Nicomp 380 (PSS, US). 

*In*-*vitro* dissolution of SRL-SMEDDS was carried out using the paddle method. Briefly, the SMEDDS containing 1 mg of SRL was filled into a hard gelatin capsule. Then 6 capsules were added into 250 mL of media stirring at 100 rpm. At each time point (15, 30, 45, 60, 90 and 120 min), 3 mL of media was collected and equal volume of fresh media was added. The concentration of SRL in the media was analyzed by the high performance liquid chromatography (HPLC; Agilent 1200, Agilent Technologies Inc, USA). Media of water, 0.4% SDS water solution, pH 1.2 hydrochloric acid solution (HCl), pH 4.5 and pH 6.8 phosphate buffer solution (PBS) were used, respectively. 

*Preparation of the SMEDDS-tablets*

To develop the SRL sustained release tablets, solid SMEDDS was first prepared. The SRL-SMEDDS was mixed with the powders of MCC and lactose by continuous grinding. The solid SMEDDS was obtained after the mixture had been incubated in a dryer overnight for complete adsorption. Tablets of the solid SMEDDS was produced using a single punch tablet machine (YPD-200C, Huanghai Co., Ltd., China). The percentage of the MCC and lactose in the solid SMEDDS was regulated to test the adsorption efficacy based on the morphology of the tablets. 

To achieve a sustained release behavior, HPMC was mixed with solid SMEDDS for tableting. The weight and hardness of the tablet were fixed at 560 mg and 60 N, respectively. Each tablet contained 1 mg of SRL. The single factor test was performed by regulating the type (100lv, K4M, K15M and K100M) and amount of HPMC. 

*In-vitro release test*

The *in-vitro* release of SRL from the tablets was carried out. Six tablets were immersed in 250 mL of water (0.4% SDS) at 37 °C. The stirring speed was 100 rpm. At each time point (2, 4, 6, 8, 10 and 12 h), 3 mL of media was collected and equal volume of fresh media was added. The concentration of SRL was determined by HPLC.

*Optimization of the tablets*

The tablets were optimized by an orthogonal experiment to achieve a proper sustained release profile. The amount of HPMC, ratio of HPMC 100lv: HPMC K4M, and amount of lactose were investigated as independent variables. The selected levels were presented in Table 1. The optimal release profile was determined to be Q_2 h _= 20%, Q_6 h _= 65% and Q_10 h _= 90% (Q represents the cumulative release) to avoid burst release and incomplete release in the initial and last stage, respectively. The release profile of the tablets was scored according to Equation 1. 

 K = |20 - Q_2 h_| + |65 - Q_6 h _| + |90 - Q_10 h_|                               Equation 1

*Characterization of the optimal tablets*

The release behavior of the SRL-SMEDDS tablets optimized by the orthogonal experiment was further evaluated. The tablets with different hardness (60, 80 and 100 N), shape (round and irregular-shaped) and preparing method (direct powder compression and wet granulation) were prepared and tested. In addition, the dissolution method (basket apparatus and paddle apparatus), stirring speed (50, 75 and 100 rpm) and dissolution medium (0.4% SDS, water, pH 1.2 HCl, and pH 6.8 PBS) were also investigated. 

Three batches of the optimized tablets were prepared and the release profiles were evaluated using the similarity factor (*f2*). When 50 < *f2* < 100 was achieved, the release profiles of the three batches of tablets would be regarded as being similar. Finally, to investigate the release kinetics, the data was plotted with the kinetic models of zero order, first order, Higuchi, and Ritger-Peppas models. 

*Pharmacokinetic study*

*Experimental design*

The pharmacokinetic study was approved by the Fuzhou General Hospital Animal Care and Use Committee. Five healthy beagle dogs (12-18 kg) were fasted but had free access to water overnight prior to the experiment. The animals were randomly divided into two groups. Each animal was orally administered with two pieces of the Rapamune^®^ or the optimal SRL-SMEDDS tablets. A crossover design with two weeks’ washout between dosing was carried out. At predetermined time intervals, 2 mL of the blood was taken from the leg veins and kept at -20 °C before analysis. 

*Quantitative analysis of SRL in blood*

The rapid resolution liquid chromatography tandem mass spectrometry (RRLC-MS/MS; Agilent, US) was used to determine the SRL blood concentration. 500 μL of blood was mixed with 500 μL of sodium acetate buffer solution (pH 4.6) and 10 μL of ascomycin (FK520) acetonitrile solution (1000 ng/mL) as internal standard. The mixture was vortexed for 30 sec and 2 mL of tert-butyl methylether was added. After being mixed in a swing mixer for 10 min, the mixture was centrifuged at 5000 rpm for 3 min. The organic layer was collected and dried under nitrogen gas at 40 °C. The mobile phase (100 μL) was added to dissolve the residues. The samples (10 μL) were then injected and separated by a ZOBAX C18 column (50 mm × 2.1 mm, 1.8 μm; Agilent, US). The column temperature was 50 °C, and water/acetonitrile (1 : 9) were used as the mobile phase at a flow rate of 0.3 mL/min. The mass spectrometer was performed using an electrospray ionization (ESI) interface in positive ionization mode and multiple reaction monitoring mode. The selected reaction monitoring of SRL and FK520 were m/z 937.2→409.3 and m/z 814.4→604.2, respectively. A standard linear calibration curve in the range of 0.5-32 ng/mL was used to determine the SRL concentration.

*Pharmacokinetic data analysis*

The maximum blood concentration (C_max_), time (T_max_) to C_max_, and area under the concentration-time curve (AUC_0−t_) were calculated based on the blood concentration versus time profiles. The relative bioavailability (Fr) of SRL-SMEDDS tablets to Rapamune^® ^was calculated using Equation 2. 

 Fr = (AUC_0→t_) _SRL - SMEDDS tablet_/(AUC_0→t_)_Rapamune_ × 100%                               Equation 2

*Statistical analysis*

The results were expressed as mean ± standard deviation. The data were analyzed by the statistical package SPSS 13.0 (SPSS Inc., US). *p *< 0.05 was considered as statistically significant. 

**Table 1:** Factors and levels of the orthogonal design

	**Factors**	**Levels**		
		**1**	**2**	**3**
A	HPMC (%)	12	15	18
B	HPMC 100lv/HPMC K4M	1:2	1:4	1:6
C	Lactose (%)	0	2	4

**Table 2:** Influence of Liquid (SMEDDS) : solid (MCC and lactose, 1 : 1) towards the appearance of tablets

**Liquid : solid**	**Appearance**
1 : 4	S
1 : 5	S
1 : 6	S
1 : 7	S
1 : 8	S
1 : 9	C

S: sticky tablets; C: clear tablets.

**Table 3:** Influence of MCC: lactose towards the appearance of tablets

**MCC : lactose**	**Appearance**
1 : 5	C
1 : 4	C
1 : 3	C
1 : 2	C
1 : 1	C
2 : 1	C
3 : 1	C
4 : 1	C

C: clear tablets.

**Table 4:** Experimental design and scores of the orthogonal array

**No.**	**Factors**			**K**
	**A (HPMC)**	**B (100lv/K4M)**	**C (Lactose)**	
1	1	1	1	23.6
2	1	2	2	22.7
3	1	3	3	20.0
4	2	1	2	0.4
5	2	2	3	8.0
6	2	3	1	7.1
7	3	1	3	36.2
8	3	2	1	48.0
9	3	3	2	58.6
K1	66.3	60.2	73.6	
K2	15.5	78.7	81.7	
K3	142.8	85.7	64.2	
R	127.3	25.5	17.5	

**Table 5:** Significance analysis of the factors affecting the score of the pellets

**Factors**	**Sum of Deviation**	**Degree of Freedom**	***F***	***P***
A	2737.576	2	23.099	0.041
B	115.722	2	0.976	0.506
C	58.389	2	0.493	0.670
Error	118.516	2		

**Table 6:** Kinetic models of the release profiles of SRL from the pellets

**Release pattern**	**Equation**	***r***
Zero order	Mt/M∞ = 0.0892t + 0.0816	0.9606
First order	ln (1-Mt/M∞) = -0.1503t + 0.6807	0.9682
Higuchi	Mt/M∞ = 0.3260t - 0.0965 1/2	0.9658
Ritger-Peppas	log (Mt/M∞) = 0.8932logt - 0.8916	0.9751

**Table 7:** Pharmacokinetic parameters after oral administration of the commercial tablets (reference) and the SRL-SMEDDS- tablets (test) (n = 6).

**Parameter**	**Test**	**Reference**
AUC0→t (ng·h/mL)	94.35 ± 21.76	88.01 ± 18.65
AUC0→∞ (ng·h/mL)	191.20 ± 81.72	112.69 ± 19.96
Tmax (h)	2.9 ± 0.2	0.9 ± 0.1
Cmax (ng/mL)	6.42 ± 1.23	13.56 ± 2.79
t1/2 (h)	54.00 ± 33.86	24.48 ± 11.17

**Figure 1:**
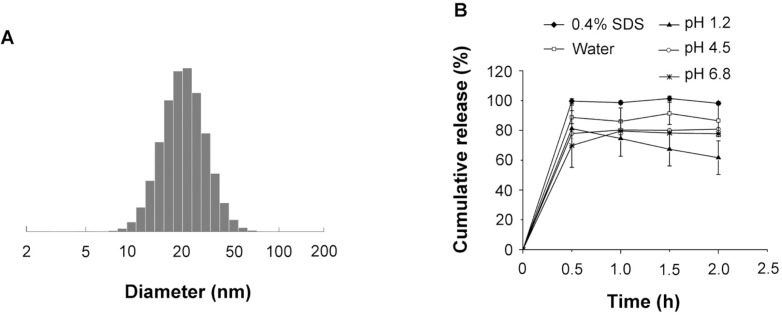
The (A) size distribution and (B) *in-vitro *dissolution of the SRL-SMEDDS in various media

**Figure 2:**
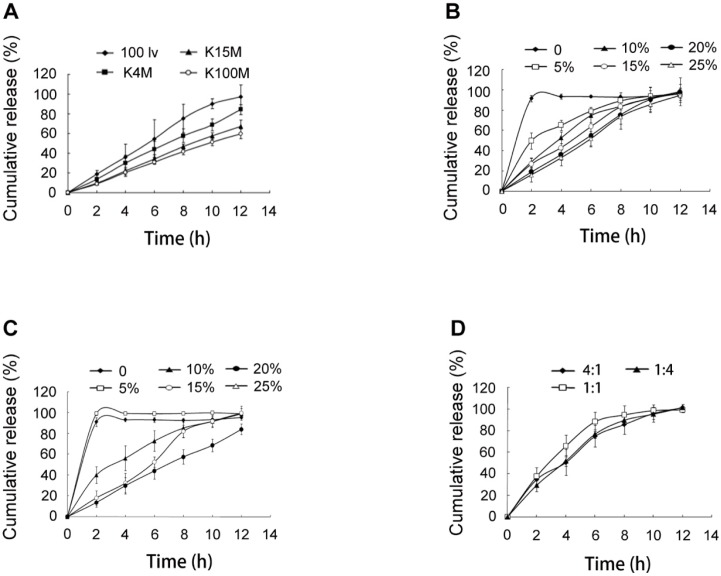
*In-vitro *release of SRL from SMEDDS-tablets. (A) tablets using HPMC of 100lv, K4M, K15M and K100M, respectively. (B) tablets using 0, 10%, 15%, 20% and 25% of HPMC 100lv. (C) tablets using 0, 10%, 15%, 20% and 25% of HPMC K4M. (D) tablets using HPMC 100lv and K4M with ratios of 4:1, 1:1 and 1:4

**Figure 3:**
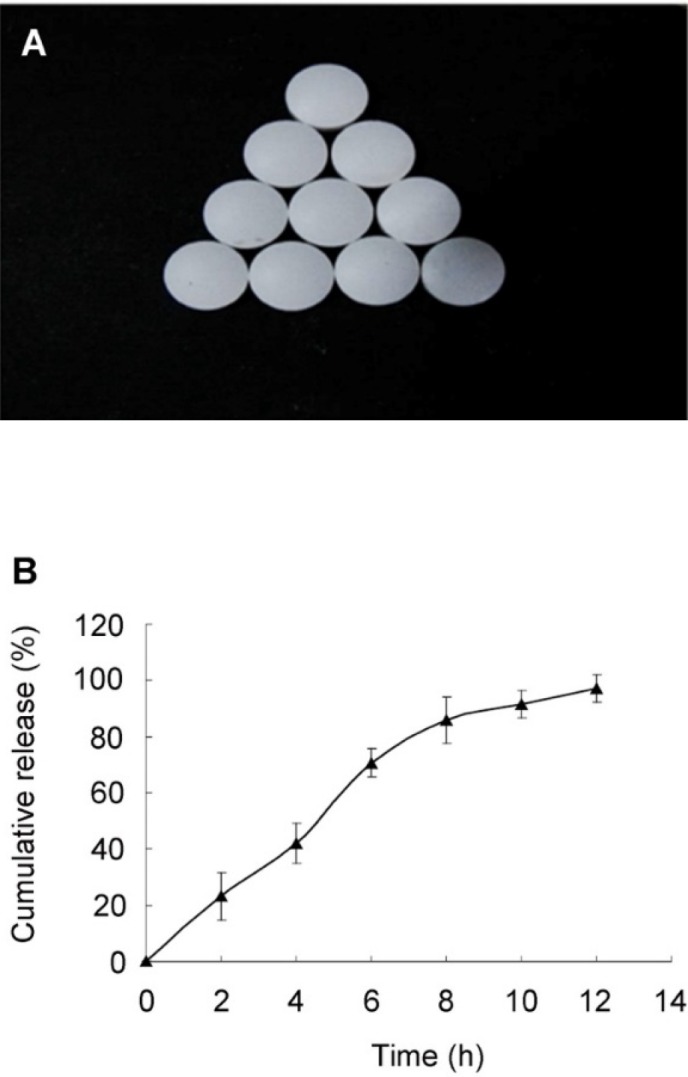
The (A) morphology and (B) *in-vitro *release of the optimal SRL-SMEDDS tablets

**Figure 4:**
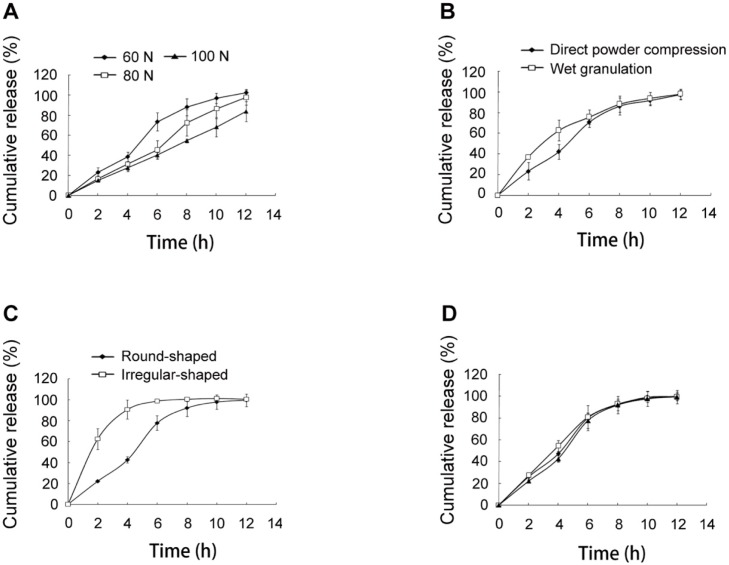
*In-vitro *release of SRL from SMEDDS-tablets. (A) tablets with hardness of 60, 80 and 100 N. (B) tablets prepared using direct powder compression and wet granulation. (C) round-shaped and irregular-shaped tablets. (D) three batches of the optimal tablets

**Figure 5:**
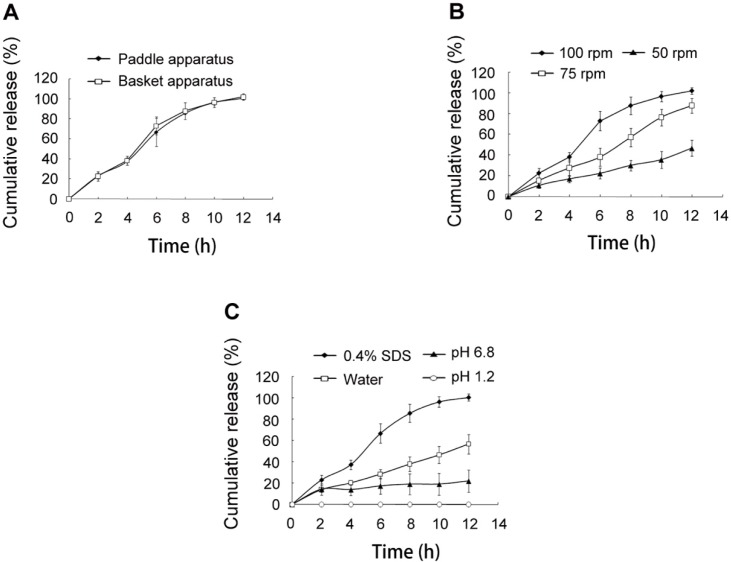
*In-vitro *release of SRL from SMEDDS-tablets. (A) release test using paddle apparatus and basket apparatus. (B) release test using stirring speed of 50, 75 and 100 rpm. (C) release test using media of water, 0.4% SDS, pH 1.2 hydrochloric acid solution, and pH 6.8 phosphate buffer solution

**Figure 6:**
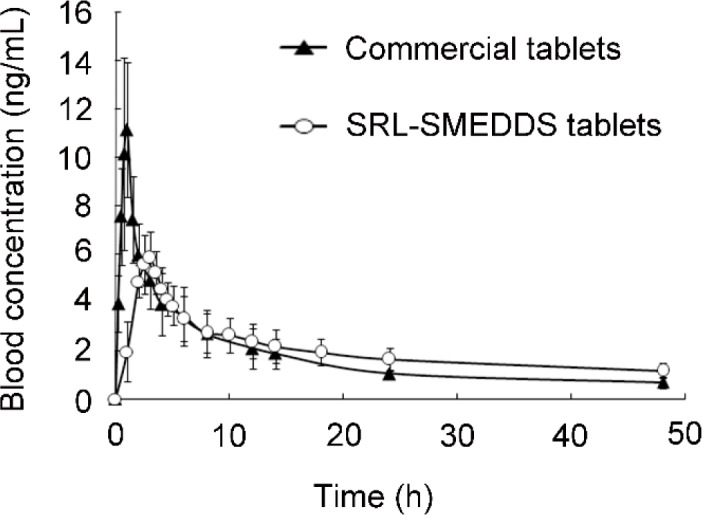
Blood concentration-time profiles of SRL after oral administration of the SRL-SMEDDS tablets and the commercial tablets (n = 5)

## Results and Discussion

*Characterization of the SRL-SMEDDS*

The average diameter of the emulsion droplets formed by the SMEDDS was 23.27 ± 0.60 nm (Figure 1A) with PDI of 0.12 ± 0.11. 

The dissolution of SRL-SMEDDS was shown in Figure 1B. In the media of 0.4% SDS water solution, over 90% of SRL was detected at 30 min. In water without SDS, the dissolution of SRL-SMEDDS was reduced, with 86.53 ± 10.72% of SRL at 2 h. The pH of the media also influenced the dissolution of SRL-SMEDDS. PBS with pH of 4.5 and 6.8 decreased the concentration of SRL, with 80.86 ± 0.78% and 77.79 ± 0.62% of SRL at 2 h, respectively. A more remarkable decrease of SRL was observed in the media of pH 1.2 HCl, with 61.69 ± 11.29% of SRL at 2 h. The degradation of SRL was caused by hydrolysis via ring opening/fragmentation in electrolyte solutions. In basic and strong acid environment, the tendency of degradation was remarkably enhanced (11). Nevertheless, the SMEDDS decelerated the speed of SRL degradation since no SRL dissolved in pH 1.2 HCl could be detected at 2 h (Supplementary file, Figure S1).

*Preparation of tablets*

SMEDDS was solidified by adsorption in MCC and lactose. As shown in Table 2, the increase of SMEDDS led to sticky tablets which was impossible for production. When the ratio of liquid to solid was 1 : 9, the sticking and picking of the tablets were disappeared. Further regulation of the ratio of MCC to lactose showed no influence on tableting (Table 3). 

The MCC and lactose had been commonly used as fillers in tablet preparation. In this study, they were also used as solid carriers of the liquid SMEDDS. Most of the SMEDDS were distributed on the surface of the solid particles, which would hamper the flow properties of the particles (12, 13).

*Single factor test*

The hydrophilic matrix system based on HPMC had been extensively explored for controlled drug delivery. In this study, HPMC 100lv, K4M, K15M, and K100M were used to obtain a sustained release of SRL. It was observed that the release speed of SRL was in the range of 100lv > K4M > K15M > K100M (Figure 2A). 

The amounts of HPMC 100lv and K4M were further investigated because of more complete release at 12 h than HPMC K15M and K100M. For both HPMC 100lv and K4M prepared tablets, the SRL release was remarkably decreased with the increase of HPMC amount (Figures 2B and 2C). When HPMC was not added, the tablets showed a rapid and complete release of 95% within 2 h. The release of SRL was significantly decelerated with 5% HPMC 100lv but a severe burst release was still existed. The release profiles of 10% and 15% HPMC 100lv prepared tablets were relatively satisfying. Addition of 20% and 25% HPMC 100lv further decreased the SRL release. A complete release of SRL at 12 h (>90%) was also observed for all the formulations. 

Similar results were found in the tablets with HPMC K4M. Differently, addition of 5% HPMC K4M had no influence on the SRL release as compared with tablets without HPMC. Further increase of HPMC reduced the release rate and 20% HPMC tablets had incomplete release (<80%) at 12 h. 

The release of SRL was largely influenced by the viscosity and amount of HPMC. The hydration of the hydrophilic HPMC polymer contributed to a viscous gel layer. The release of the drugs was realized by dissolution and diffusion through the gel network. Gradual erosion of the gel layer also contributed drug release (14). Higher viscosity and amount of HPMC resulted in increased density of the gel network, and prolonged period for the swelling and erosion of the gel layer (15). As a consequence, the release of SRL from the tablets was decelerated with the increase of viscosity and amount of HPMC. 

The HPMC 100lv showed more satisfying release profiles than HPMC K4M. However, it was also found that the reproducibility was poor when the amount of HPMC 100lv was less than 15% due to rapid swelling and erosion of the tablets.

Combination of two viscosity grades of HPMC could get a desired apparent viscosity (16). Thus, the HPMC 100lv and HPMC K4M were combined to prepare tablets. With a total of 15% HPMC, the amount of HPMC 100lv had a negative influence on the initial release, showing a burst release at 2 h (Figure 2D). It was also observed that the tablets with equal amount of HPMC 100lv and HPMC K4M had the most rapid release due to fast swelling and erosion of HPMC 100lv.

*Optimization of the tablets*

An L9(3^4^) orthogonal experiment was used to optimize the release profile of the tablets (Table 4). The influence of the amount of HPMC (A), ratio of HPMC 100lv : HPMC K4M (B), and amount of lactose (C) towards the SRL release was in the range of A > B > C. As shown in Table 5, difference (*p* = 0.041) was found among the three levels of variable A while variable B and C showed no significant difference (*p* > 0.05). Since the MCC had a porous structure and more SMEDDS could be adsorbed than the lactose, lactose was removed from the formulation. The optimal combination would be A2B1C1, with 15% HPMC (100lv : K4M = 1 : 2). The rest of the tablets was filled with MCC as filling agent. The tablets based on the optimal formulation had smooth surface and complete shape (Figure 3A). A controlled and complete release was achieved, with 23.02 ± 3.00%, 66.73 ± 14.21%, and 96.50 ± 2.63% of SRL at 2, 6, 10 h, respectively (Figure 3B).

*Characterization of the tablets*

*Hardness*

The release of SRL was reduced by regulating the hardness of the tablets from 60 N to 80 N and 100 N (Figure 4A). 

The increased compression pressure led to smaller size of the micropores in the tablets, which further decelerated the diffusion of water through the pores (17). As a result, the hydration of HPMC and dissolution of SRL were delayed.

*Preparing method*

The preparing method of the tablets was also compared. As shown in Figure 4B, the SRL release was more rapid in the initial 4 h in the tablets with wet granulation than those with direct compression. For the wet granulation method, 90% ethanol was used as binder. During the drying process, the binder in the surface of the tablets was first evaporated. Continuing evaporation of ethanol from the inside to the outside was accompanied with the migration of SMEDDS to the surface of the tablets. Therefore, the tablets with the wet granulation method had a more obvious burst release. 

*Shape*

The shape of the tablets was compared. Based on the optimal formulation, irregular-shaped tablets were prepared with the same method and hardness as the round-shaped tablets. A remarkable burst release of over 60% at 2 h was observed (Figure 4C). The reason might be that the irregular-shaped tablets had higher surface area, which accelerated the swelling and erosion of the tablets. 

*Repeatability*

Three batches of the optimal tablets were prepared (Figure 4D). The *f*2 values of the release curves were 74, 76, and 72, indicating excellent similarity of the release behavior of the tablets. Thus, the optimal tablets achieved favorable repeatability.

*Release method*

The basket apparatus and paddle apparatus were used for the release test, respectively. It was found that the release profiles were similar (Figure 5A), indicating that the release method had no influence on the release of SRL. 

*Stirring speed*

When the release test was carried out using reduced stirring speed of 75 rpm and 50 rpm, the SRL release was greatly reduced (Figure 5B). The stirring speed was used to simulate gastrointestinal motility of different aged people. Higher speed resulted in more rapid and complete release of SRL from the tablets. The vortex caused by stirring accelerated the hydration and erosion of HPMC. 

*Medium*

The *in-vitro* release was performed in various media (Figure 5C). The release speed was obviously decreased in water without SDS, which might be caused by the nonsink environment. Less than 30% of SRL was detected in pH 6.8 PBS buffer solution. The nonsink environment limited the SRL release speed. Meanwhile, the pH had a negative influence on the stability of SRL as the hydrolysis of SRL was enhanced in pH 6.8 PBS as compared with water (Figure S1). No SRL was detected in the media of pH 1.2, in which SRL had a huge tendency of hydrolysis (11). Though SMEDDS improved the pH stability of SRL by reducing the chances of SRL contacting the media, the degradation of SRL in the emulsion droplets was still remarkable because of incubating as long as 12 h. 

*Release kinetic*

Modeling of the release profile of the optimal tablets was investigated. The linear correlation *r *was used to determine the best fit kinetic model. As shown in Table 6, the Ritger-Peppas model had the largest value of *r *= 0.9751, suggesting that the SRL release was best explained by the Ritger-Peppas model. The value of the constant (*n*) was 0.8932, indicating that the main mechanism of the drug release was erosion.

*Pharmacokinetic study*

The blood concentration-time curve of SRL was shown in Figure 6. Detailed profiles were presented in Table 7. Compared with Rapamune^®^, the self-prepared tablets had decreased C_max_ (6.42 ± 1.23 ng/mL *vs.* 13.56 ± 2.79 ng/mL, *p* < 0.05). Thus, the SRL-SMEDDS tablets reduced the tendency of SRL blood concentration exceeding the therapeutic range, contributing to better safety. The SRL-SMEDDS tablets also showed increased T_max_ (2.9 ± 0.2 h *vs.* 0.9 ± 0.1 h, *p* < 0.05) and t_1/2_ (54.00 ± 33.86 h^-1 ^*vs.* 24.48 ± 11.17 h^-1^, *p* > 0.05), suggesting that the SRL-SMEDDS tablets maintained more steady blood concentration than the commercial tablets.

The AUC of the SRL-SMEDDS tablets and Rapamune^®^ was 94.35 ± 21.76 ng·h/mL and 88.01 ± 18.65 ng·h/mL (*p *> 0.05), respectively. The relative bioavailability was 107.20%, indicating that the self-prepared tablets were bio-equivalent to the commercial tablets. *In-vitro* degradation of SRL was rapid in the strong acid media while SMEDDS improved the stability of SRL, and the gastric emptying guaranteed a relatively short incubating period. Though degradation of SRL was also found in pH 6.8 with less than 80% left at 12 h, the controlled release property and fast absorption of SRL-SMEDDS minimized the percentage of degraded SRL. 

Our previous study showed that the SRL-SMEDDS had improved bioavailability upon Rapamune^® ^(9). Preparation of sustained release system, however, might decrease the bioavailability because of incomplete release *in-vivo* and diverse absorption efficacy in different section of the intestinal (18). 

## Conclusion

In this study the SRL-SMEDDS tablets were developed. HPMC 100lv and HPMC K4M were combined to achieve a sustained release of SRL. The release behavior was optimized by an orthogonal experiment. The influences of hardness, shape, and preparing method of tablets towards the *in-vitro* release were investigated. The optimal tablets were further tested for release method, stirring speed, and medium. The release kinetic was demonstrated to be the best fit of the Ritger-Peppas model with a mechanism of erosion. The SRL-SMEDDS tablet was bio-equivalent to the commercial tablet but it had decreased C_max_ and more steady blood concentration. In conclusion, this study provided a promising candidate of SRL with a sustained release behavior and better safety. 
